# Epidemiology, management, and outcome of infection, sepsis, and septic shock in a German emergency department (EpiSEP study)

**DOI:** 10.3389/fmed.2022.997992

**Published:** 2022-10-17

**Authors:** Nicole Wolfertz, Lennert Böhm, Verena Keitel, Oliver Hannappel, Philipp Kümpers, Michael Bernhard, Mark Michael

**Affiliations:** ^1^Emergency Department, Medical Faculty, University Hospital of Düsseldorf, Heinrich Heine University Düsseldorf, Düsseldorf, Germany; ^2^Clinic for Gastroenterology, Hepatology and Infectious Diseases, Medical Faculty, University Hospital of Düsseldorf, University of Magdeburg, Magdeburg, Germany; ^3^Informations-, Kommunikations- und Medizintechnik (IKMT), University Hospital of Düsseldorf, Düsseldorf, Germany; ^4^Division of General Internal and Emergency Medicine, Nephrology, Hypertension and Rheumatology, Department of Medicine D, Münster University Hospital, Münster, Germany

**Keywords:** epidemiology, infection, sepsis, septic shock, emergency department

## Abstract

**Background:**

The adjacent conditions infection, sepsis, and septic shock are among the most common causes of treatment in the emergency department (ED). Most available data come from intensive care units (ICU) and include nosocomial infections acquired during hospitalization. Epidemiological data from German EDs are not yet available, although the ED is one of the first points of contact for patients. The aim of this study was to investigate the epidemiology, causes, diagnosis, mortality, and treatment of patients with infections in the ED.

**Materials and methods:**

In this retrospective, single-center observational study, routinely collected data from the patient data management system and from the hospital information system were analyzed. All adult patients who presented to the ED in connection with an infection during the study period from 01/01 to 28/02/2019 were included. Exclusion criteria were age ≤ 17 years and incomplete records. Three groups (I. Infection, II. Sepsis, and III. Septic shock) were defined according to SEPSIS-3 definitions.

**Results:**

During the study period, a total of 6,607 patients were treated in the ED. Of these patients, 19.3% (*n* = 1,278) had an infection (mean age 56 ± 23 years, 50% female). The sites of infection were distributed as follows: Respiratory tract 35%, genitourinary tract 18%, maxillofacial/ears/nose/throat 14%, intraabdominal 13%, soft tissues 10%, central nervous system 1%, other cause 3%, or unknown cause 6%. Infection only, sepsis and septic shock were present in 86, 10, and 3%, respectively. There were significant differences in vital signs as well as in the various emergency sepsis scores across the predefined groups [I vs. II vs. III: SOFA (pts.): 1 ± 1 vs. 4 ± 2 vs. 7 ± 3 (*p* < 0.0001), systolic blood pressure (mmHg): 137 ± 25 vs. 128 ± 32 vs. 107 ± 34 (*p* < 0.05), heart rate (bpm): 92 ± 18 vs. 99 ± 23 vs. 113 ± 30 (*p* < 0.05), respiratory rate (min-1): 18 ± 4 vs. 20 ± 7 vs. 24 ± 10 (*p* < 0.05)]. In the three groups, blood cultures were obtained in 34, 81, and 86%, of cases, respectively and antibiotics were administered in the ED in 50, 89, and 86%, of cases respectively. The 30-day mortality rate in the three groups was 1.6, 12.0, and 38.1%, respectively.

**Conclusion:**

This study is the first to show the incidence, management, and outcome of patients classified as infection, sepsis, and septic shock in a German ED. The findings of our real-world data are important for quality management and enable the optimization of treatment pathways for patients with infectious diseases.

## Introduction

Infections, sepsis, and septic shock are among the most common causes of treatment in the emergency department (ED) (1–3). The recognition, diagnosis, and initial treatment of patients with infections, sepsis, and septic shock represent a challenge for all involved in medical care that should not be underestimated (4, 5). For this reason, there are international and national recommendations for the management of sepsis (6–8).

However, while local infection can be treated well in the ED, patients with sepsis and the associated life-threatening organ dysfunction show a considerably less favorable course of disease associated with high mortality (1, 2, 4, 9).

Therefore, it is relevant to know the source of the most common infections, sepsis, and septic shock even in the ED (10). It is important to note that the source and frequency of infectious diseases treated in the ED do not necessarily have to correspond to those found in sepsis or septic shock. Compared to the quality of national and international epidemiological knowledge from the Intensive Care Unit (ICU) data on infection, sepsis and septic shock in the ED are extremely sparse (6).

The collection of appropriate real-life data is the basis for future optimization of training and care concepts, early detection, development of guidelines and standard operating procedures (SOP), and patient safety in the ED. The aim of this retrospective, single-center study was therefore to compare epidemiology, management, and outcome of patients with infection, sepsis, or septic shock in a German ED.

## Materials and methods

### Study design and population

In this retrospective, mono-centric observational study, all adult patients admitted to the ED of the University Hospital Düsseldorf for infection, sepsis, or septic shock between 01/01 and 28/02/2019 were included. The study protocol was approved by the Ethics Committee of the Medical Faculty of the University of Düsseldorf, Germany (2020-973).

### Setting

More than 44,000 patients are treated annually in the ED of the University Hospital of Düsseldorf, Germany. The ED is the first point of contact for almost all non-scheduled emergency patients. Only patients requiring urgent intervention (e.g., ST-segment elevation infarct) bypass the ED according to local protocols. The ED is part of a level I trauma center for the treatment of severely injured patients by a dedicated trauma team in accordance with national recommendations (11). Out-of-hospital care is provided by a two-tier emergency medical service (EMS) staffed with paramedics and emergency physicians. At our facility, patients are cared for in the ED by a team of nurses, residents, and senior physicians with expertise in emergency and critical care medicine. There are twelve regular cabins, four resuscitation rooms and a decision unit with twelve monitored beds in the ED.

### Data collection

Demographical and medical care data were anonymously aggregated from the patient data management system (COPRA^®^, COPRA System GmbH, Berlin, Germany) and the hospital information system (MEDICO^®^, Cerner Deutschland GmbH, Itstein, Germany) by database query and transferred to a spreadsheet program (Microsoft^®^ Office 365, version 16.37, Microsoft Corporation, Redmond, WA, USA). The analysis included age, sex, weight, height, infectious diseases, comorbidities, site of infection, in-hospital treatment (e.g., fluid resuscitation, laboratory tests, blood cultures, antibiotic therapy, therapeutic measures), vital signs (e.g., systolic blood pressure, respiratory rate, oxygen saturation by pulse oximetry, body temperature), transfer location (e.g., normal ward, ICU), and outcomes (length of stay in the ED, length-of-hospital-stay, 30-day mortality). The time of measurement of the data evaluation in relation to the 1 h-bundle refers to 1 h after admission to the ED.

### Study definitions and emergency medicine sepsis scores

Patients were divided into three groups: I: Infection alone (without sepsis or septic shock), II: sepsis, III: septic shock based on the current SEPSIS-3 definition (6). Accordingly, sepsis is defined as a life-threatening organ dysfunction due to dysregulation resulting from infection. Organ dysfunction is determined by an acute 2-point change in SOFA score (6, 12). Septic shock is defined by catecholamine requirement to maintain a mean arterial blood pressure (MAP) of above 65 mmHg and a lactate level greater than 2 mmol/L despite adequate volume substitution (30 ml/kg) (6).

The following scores were calculated for all patients enrolled: Quick Sequential Organ Failure Assessment score (qSOFA) (6), SIRS (13), Prehospital Early Sepsis Detection (PRESEP) (14), modified National Early Warning Score (MEWS) (15), Sepsis-related organ failure assessment (SOFA) (12), and Mortality in Emergency Department Sepsis (MEDS) (16).

### Statistical analysis

Data are presented as numbers and percentage, mean ± standard deviation (SD), median with interquartile ranges, as appropriate. The chi-squared test was applied for categorical data, and the Student’s *t*-test for metric data. All tests used were two-sided, and statistical significance was set at *p* < 0.05. Microsoft Excel 2011 (Microsoft, Redmond, WA, USA) and DataGraph 4.5.1 (Visual Data Tools Inc. 2006–2022) were used for statistical analyses and to prepare figures.

## Results

**FIGURE 1:**
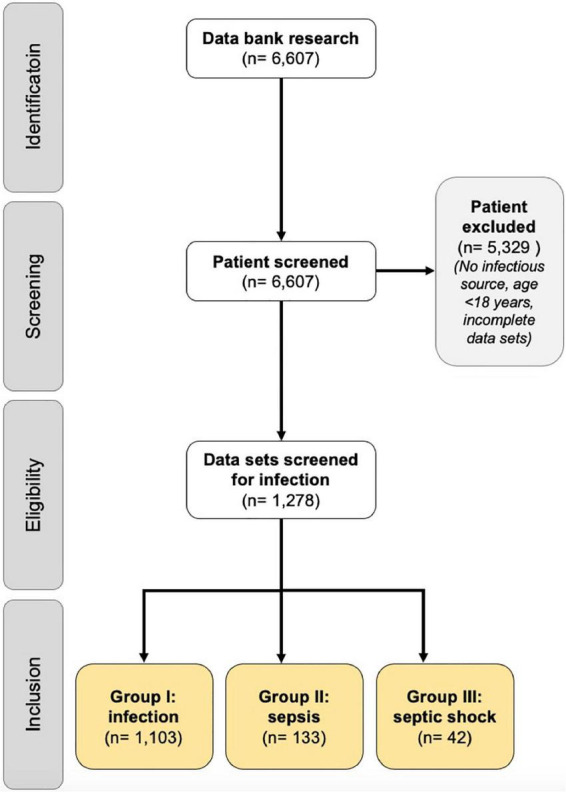
Prisma chart. A total of 6,607 patients were detected from the patient management system database as a potential study population. These 6,607 patients were screened for inclusion and exclusion criteria, resulting in the inclusion of 1,278 patients with documented infection focus. A total of 5,329 patients had to be excluded from the study because of relevant exclusion criteria (e.g., age < 18 years. Incomplete records, no infection focus). The study population of 1,278 patients was divided into infection (group I), sepsis (group II), and septic shock (group III) groups.

During the 2-month study period, a total of 6,607 patients of all ages were treated in the ED. Patients with incomplete records and patients ≤ 17 years were excluded. After detailed screening and individual case examination, the final data set consisted of 1,278 patients (19.3%) with an infectious disease. Of these patients, 1,105 (86.5%) patients had an infection (group I), 133 (10.4%) had sepsis (group II), and 42 (3.3%) had septic shock (group III). The participant flow chart is shown in Figure 1. In relation to all ED visits during the study period, these results correspond to an incidence of infection alone, sepsis and septic shock of 16.7, 2.0, and 0.6%, respectively.

### Patient characteristics

An overview of patient characteristics is provided in Table 1. The age of the patients increased significantly across the three predefined groups. In comparison to group I, the frequency of pre-existing concomitant conditions and life-limiting comorbidities, increased in groups II and III. The different emergency medicine sepsis scores steadily increased across the three predefined groups (Table 1).

**TABLE 1:** Patient characteristics of the patients suffered from infection, sepsis, and septic shock in the emergency department.

	All (*n* = 1,278)		Group I infection (*n* = 1,103)		Group II sepsis (*n* = 133)		Group III septic shock (*n* = 42)	
Age (years, mean ± SD)	56 ± 23		53 ± 23*		69 ± 19**		72 ± 13	
Male sex [*n* (%)]	637 (49.8)		546 (49.5)		67 (50.4)		25 (59.5)	
**Hospital admission by**								
EMS [*n* (%)]	520 (40.7)		390 (35.4)*		92 (69.2)**		38 (90.5)***	
Walking emergency [*n* (%)]	600 (46.9)		568 (51.5)*		29 (21.8)**		3 (7.1)***	
Family doctor [*n* (%)]	91 (7.1)		88 (8.0)*		3 (2.3)		0 (0.0)	
MET [*n* (%)]	25 (2.0)		20 (1.8)		4 (3.0)		1 (2.4)	
Interhospital transfer [*n* (%)]	13 (1.0)		10 (0.9)		3 (2.3)		0 (0.0)	
Medical specialist [*n* (%)]	29 (2.3)		27 (2.4)		2 (1.5)		0 (0.0)	
**Residence**								
Home [*n* (%)]	1167 (91.3)		1023 (92.7)*		111 (83.5)		33 (78.6)***	
Nursing home [*n* (%)]	110 (8.6)		79 (72)*		22 (16.5)		9 (21.4)***	
**Pre-existing conditions**								
COPD [*n* (%])	119 (9.3)		85 (7.7)*		22 (16.5)		12 (28.6)***	
Kidney disease [*n* (%)]	164 (12.8)		114 (10.3)*		39 (29.3)		11 (26.2)***	
with dialysis [*n* (%)]	42 (3.3)		21 (1.9)*		16 (12.0)		5 (11.9)***	
Heart failure [*n* (%)]	54 (4.2)		42 (3.8)*		11 (8.3)		1 (2.4)	
Immunosuppression [*n* (%)]^1^	149 (11.7)		115 (10.4)*		26 (19.5)		8 (19.0)	
Liver cirrhosis [*n* (%)]	19 (1.5)		13 (1.2)*		5 (3.8)		1 (2.4)	
Diabetes mellitus [*n* (%)]	207 (16.2)		153 (13.9)*		39 (29.3)		15 (35.7)***	
with insulin [*n* (%)]	81 (6.3)		63 (5.7)		13 (9.8)		5 (11.9)	
Malnutrition [*n* (%)]	34 (2.7)		23 (2.1)		6 (4.5)		5 (11.9)***	
Tumor disease [*n* (%)]	133 (10.4)		100 (9.1)*		22 (16.5)		11 (26.2)***	
Chemo-/Radiotherapy [*n* (%)]	49 (3.8)		38 (3.4)		9 (6.8)		2 (4.8)	
Hematological diseases [*n* (%)]	126 (9.9)		85 (7.7)*		31 (23.3)		10 (23.8)***	
Transplantation [*n* (%)]	53 (4.1)		40 (3.6)*		10 (7.5)		3 (7.1)	
HIV [*n* (%)]	13 (1.0)		11 (1.0)		2 (1.5)		0 (0.0)	
None [*n* (%)]	731 (57.2)		695 (63.0)*		29 (21.8)		7 (16.7)***	
**Laboratory values**								
Creatinine (mg/dl, median, IQR)	0.92 (0.73–1.26)		0.87 (0.71–1.12)*		1.41 (0.96–2.15)		1.67 (1.14–3.01)***	
Bilirubine (mg/dl, median, IQR)	0.50 (0.34–0.79)		**0.47 (0.33–0.74)**		0.63 (0.44–1.06)		0.62 (0.37–1.05)***	
Leucocyts (X1000/μl, median, IQR)	10.2 (7.40–14.05)		10.0 (7.4–13.73)*		11.7 (6.80–14.60)		12.8 (10.10–17.73)***	
Thrombocyts (X1000/μl, median, IQR)	245 (189–306)		250 (200–307)*		186 (141–285)		225 (166.25–277.50)***	
Lactate (mmol/l, median, IQR)	1.5 (1.10–2.10)		1.4 (1.00–2.00)*		2.0 (1.30–2.50)**		3.5 (1.68–5.45)***	

**Vital signs (median, IQR)**	**admission**	**discharge**	**admission**	**discharge**	**admission**	**discharge**	**admission**	**discharge**

SBP (mmHg; median, IQR)	133 (119–147)	119 (103–141)	134 (122–148)*	124 (107–145)*	123 (109–144)**	110 (99–139)	100 (86–132)	102 (94–134)***
HR (bpm; median, IQR)	92 (80–105)	82 (72–96)	90 (80–102)*	80 (70–92)*	99 (87–102)**	86 (71–103)**	110 (97–125)***	91 (80–123)***
SI (min/mmHg; median, IQR)	0.7 (0.6–0.8)	0.7 (0.6–0.9)	0.7 (0.6–0.8)*	0.6 (0.5–0.8)*	0.8 (0.7–1.0)**	0.8 (0.6–1.0)**	1.1 (0.8–1.4)***	**0.9 (0.8–1.0)**
SpO2 (%,; median, IQR)	97 (95–99)	96 (94–98)	97 (95–99)	96 (94–98)	96 (93–98)	96 (94–99)**	94 (91–98)***	95 (93–99)***
RR (min-1; median, IQR)	18 (15–20)	22 (18–27)	18 (15–19)*	22 (18–27)	18 (16–21)*	23 (19–28)	20 (16–29)***	22 (17–27)
Temp (°C; median, IQR)	36.9 (36.3–37.3)	37 (36.3–37.8)	36.9 (36.4–37.6)	37 (36.4–38)	36.9 (36.2–38)	37.3 (36.4–37.8)	36.6 (36–38)	36.2 (35.7–37.5)
GCS (points; median, IQR)	15 (15–15)	n.a.	15 (15–15)*	n.a.	15 (14–15)**	n.a.	14 (9–15)***	n.a.
**Emergency medicine Sepsis scores**								
qSOFA (pts; median, IQR)	0 (0–0)		0 (0–0)*		1 (0–1)		1 (1–2)***	
SOFA (pts; median, IQR)	1 (0–2)		0 (0–1)*		3 (2–5)**		7 (5–8)***	
SIRS (pts; median, IQR)	1 (1–2)		1 (0–2)*		2 (1–3)		2 (2–3)***	
MEWS (pts; median, IQR)	1 (1–3)		1 (0–2)*		3 (1–4)**		5 (3–6)***	
PRESEP (pts; median, IQR)	2 (0–4)		2 (0–3)*		3 (2–5)**		5 (3–6)***	
MEDS (pts; median, IQR)	3 (0–8)		3 (0–6)*		8 (5–11)**		13 (10–15)***	

*N*, number; SD, standard deviation; pts, points; RSV, respiratory syncytial virus; EMS, emergency medical services; MET, medical emergency team; COPD, chronic obstructive pulmonary disease; HIV, human immunodeficiency virus; SBP, systolic blood pressure; HF, heart rate; bpm, beats per minute; SI, shock index; SpO2, oxygen saturation by pulse oximetry; RR, respiratory rate; Temp, temperature tympanal; GCS, Glasgow Coma Scale; IQR, interquartile range; qSOFA, quick sequential organ failure assessment; SOFA, sepsis-related organ failure assessment score; SIRS, systemic inflammatory response syndrome; MEWS, modified early warning score; PRESEP, prehospital early sepsis detection score; MEDS, mortality in emergency department sepsis score.

*p* is significant, if *p* < 0.05; * = *p*_I,II_ < 0.05; ** = *p*_II,III_ < 0.05; *** = *p*_I,III_ < 0.05.

The bold values represent significant results.

### Vital signs

**FIGURE 2:**
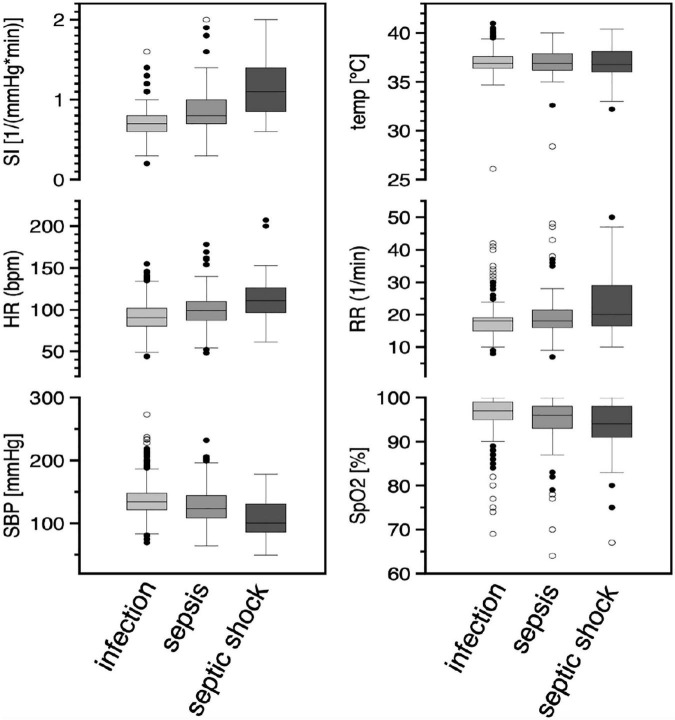
Vital signs in the three subgroups of patients. Results were illustrated as box-and-whisker-plot with 25 and 75%-quantiles (box) median (bar), 1.5X interquartile range and outliers (circles) in the three subgroups (I: infection *n* = 1,103, II: sepsis *n* = 133, III: septic shock *n* = 42). Abbreviations: SI, shock index; HR, heart rate; SBP, systolic blood pressure; temp, temperature tympanal; RR, respiratory rate; SpO2, oxygen saturation by pulse oxymetry.

The variability of vital signs in the three groups is shown in Figure 2. The shock index increases with the severity of the disease, with increasing tachycardia and hypotension (Table 1). Meanwhile, the variability of the measured body temperature and respiratory rate increases (Table 1).

### Source of infection

**FIGURE 3:**
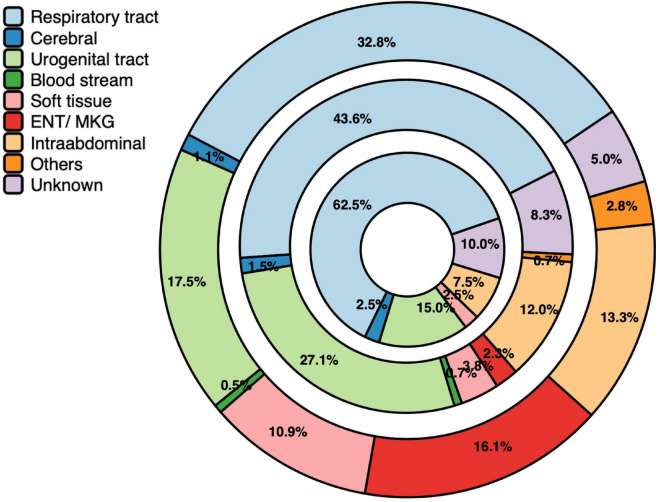
Sources of infection. Results were illustrated as circles represented the distribution in percentage (%) of the infection sites in the three subgroups (I: infection *n* = 1,103, outer circle; II: sepsis *n* = 133, middle circle; III: septic shock *n* = 42, inner circle).

The sources of infection in the groups I–III are shown in Figure 3. The predominant site of infection in all groups was the respiratory tract. Compared to group I, the proportion of respiratory infections doubled in patients with septic shock. The second most common source of infection was the genitourinary tract Infections of soft tissue, maxillofacial/ears/nose/throat were predominantly found in group I, suggesting a less frequent cause of sepsis and septic shock. The proportion of intra-abdominal infections decreased slightly with increasing sepsis severity from 13 to 9.5%. Despite extensive diagnostic investigations, the source of infection remained elusive in 5.0, 8.3, and 9.5% of patients in group I, II, and III, respectively.

### Diagnostic workup and emergency interventions

Diagnostic procedures performed during treatment in the ED are presented in Table 2. The distribution of focus in the three groups was also reflected in the samples collected in the ED. Urine status, and, in the case of sepsis or septic shock, urine culture was obtained most frequently. With increasing severity of the disease, the diagnostic effort for the focus search increased significantly. Similar to the predominant respiratory source of infections, point-of-care testing (POCT) for influenza A/B and RSV was also performed in all three groups.

**TABLE 2:** Diagnostic workup performed in the emergency department.

	All (*n* = 1,278)	Group I infection (*n* = 1,103)	Group II sepsis (*n* = 133)	Group III septic shock (*n* = 42)
**Sampling**				
Sputum [*n* (%)]	16 (1.3)	9 (0.8)*	4 (3.0)	3 (7.1)***
Urine [*n* (%)]	672 (52.6)	541 (49.0)*	100 (75.2)	31 (73.8)***
Urine culture [*n* (%)]	340 (26.6)	251 (22.8)*	69 (47.6)	20 (47.6)***
Stool sample [*n* (%)]	29 (2.3)	21 (1.9)*	7 (5.3)	1 (2.4)
Drain secretion [*n* (%)]	24 (1.9)	15 (1.4)*	6 (4.5)	3 (7.1)***
Liquor [*n* (%)]	11 (0.9)	7 (0.6)	2 (1.5)	2 (4.8)***
POCT Influenza A/B, RSV [*n* (%)]	403 (31.5)	320 (29.0)*	63 (47.4)	20 (47.6)***
**Imaging**				
Chest x-ray [*n* (%)]	558 (43.7)	421 (38.2)*	102 (76.7)	35 (83.3)***
TTE [*n* (%)]	194 (15.2)	134 (12.1)*	42 (31.6)	18 (42.9)***
Abdominal sonography [*n* (%)]	351 (27.5)	257 (23.3)*	71 (53.4)	23 (54.8)***
Computed tomography [*n* (%)]	249 (19.5)	180 (16.3)*	45 (33.8)**	24 (57.1)***
**Antibiotic therapy**				
Oral [*n* (%)]	262 (20.5)	255 (23.1)*	7 (5.3)	0 (0.0)***
Intravenous [*n* (%)]	441 (34.5)	294 (26.7)*	111 (83.5)	36 (85.7)***
After blood cultures [*n* (%)]	420 (32.9)	294 (26.7)*	94 (70.7)	32 (76.2)***
**Compliance to 1-h bundle^#^**				
Fluid resuscitation [*n* (%)]	28 (2.2)	12 (1.1)*	9 (6.8)**	7 (16.7)***
Laboratory investigation [*n* (%)]	1124 (88.1)	952 (86.3*)	131 (98.5)	441 (97.6)***
Blood cultures [*n* (%)]	518 (40.5)	374 (33.9)*	108 (81.2)	36 (87.8)***
Lactate measurement [*n* (%)]	940 (73.6)	776 (70.4)*	126 (94.7)	38 (95.0)***
Vasopressor for MAP ≥ 65 [*n* (%)]	39 (3.1)	0 (0.0)*	4 (3.0)**	35 (83.3)***
Antibiotic therapy within 1 h [*n* (%)]	75 (5.9)	58 (5.3)	11 (8.3)	6 (14.3)***
**Oxygen therapy**				
Mask [*n* (%)]	83 (6.5)	48 (4.4)*	22 (16.5)**	13 (30.9)***
CPAP/NIV [*n* (%)]	5 (0.4)	4 (0.4)	0 (0.0)	1 (2.4)
Invasive ventilation [*n* (%)]	12 (0.9)	2 (0.2)*	3 (2.3)**	7 (16.7)***
**Instrumentation**				
Central venous access [*n* (%)]	55 (4.3)	11 (1.0)*	11 (8.3)**	33 (78.6)***
Arterial line [*n* (%)]	74 (5.8)	15 (1.5)*	23 (17.3)**	36 (85.7)***
Catecholamines [*n* (%)]	39 (3.1)	0 (0.0)*	4 (3.0)**	35 (83.3)***

*N*, number; POCT, point of care testing; RSV, respiratory syncytial virus; ECG, electrocardiogram; TTE, transthoracic echocardiography; MAP, mean arterial blood pressure; CPAP/NIV, continuous positive airway pressure/non-invasive ventilation.

^#^Time of measurement: 1 h after admission to the emergency department.

*p* is significant, if *p* < 0.05; * = *p*_I,II_ < 0.05; ** = *p*_II,III_ < 0.05; *** = *p*_I,III_ < 0.05.

The bold values represent significant results.

While only 16.3% of patients in group I underwent computed tomography (CT), CT was performed in 57.1% of patients in group III. Also, the use of sonography increased from 23% in group I to 53% in group II to 55% in group III.

As expected, the frequency of antibiotics administered orally decreased from 23.1 to 5.3% and 0% in groups I, II, and III, respectively. The opposite was observed for the frequency of administration of intravenous antibiotics within the first hour after admission to the ED (groups I-III: 5.3, 8.3, and 14.3%, respectively).

Guideline-based therapy according to the 1-h bundle and other ED emergency interventions increased in patients with sepsis and septic shock (Table 2).

### Relocation sites and outcomes

Cohort-specific relocations sites and outcomes are shown in Table 3. Significant differences for the three groups are evident in the subsequent follow-up treatment. While 51% of patients from group I can still be treated as outpatients, the respective percentage in group III is 0%. Conversely, 1.5% from group I, 14.3% from group II, and 59.5% from group III required intensive care.

**TABLE 3:** Relocations sites and outcomes of the patients suffered from infection, sepsis, and septic shock in the emergency department.

	All (*n* = 1,278)	Group I infection (*n* = 1,103)	Group II sepsis (*n* = 133)	Group III septic shock (*n* = 42)
**Primary relocation site after ED**				
Intensive care unit [*n* (%)]	61 (5.5)	17 (1.5)*	19 (14.3)**	25 (59.5)***
Stroke unit [*n* (%)]	14 (1.1)	10 (0.9)	3 (2.3)	1 (2.4)
Intermediate Care [*n* (%)]	10 (0.8)	8 (0.7)	2 (1.5)	0 (0.0)
Catheter laboratory [*n* (%)]	4 (0.3)	2 (0.2)	1 (0.8)	1 (2.4)***
Operation theater [*n* (%)]	11 (0.9)	8 (0.7)	2 (1.5)	1 (2.4)
Normal ward [*n* (%)]	500 (39.1)	402 (36.4)*	95 (71.4)**	3 (7.1)***
Interhospital transfer [*n* (%)]	41 (3.2)	30 (2.8)	5 (3.8)**	6 (14.3)***
Discharge at home [*n* (%)]	569 (44.5)	566 (51.2)*	3 (2.3)	0 (0.0)***
Discharge against medical aid [*n* (%)]	41 (3.2)	40 (3.6)	1 (0.8)	0 (0.0)
Other outpatient clinics [*n* (%)]	21 (1.6)	20 (1.8)	1 (0.8)	0 (0.0)
**Outcomes**				
Admission to				
ICU [*n* (%)]	61 (5.5)	17 (1.5)*	19 (14.3)**	25 (59.5)***
Normal ward [*n* (%)]	500 (39.1)	402 (36.4)*	95 (71.4)**	3 (7.1)***
Interhospital transfer [*n* (%)]	41 (3.2)	30 (2.8)	5 (3.8)**	6 (14.3) ***
Discharge at home [*n* (%)]	569 (44.5)	566 (51.2) *	3 (2.3)	0 (0.0) ***
Death in ED [*n* (%)]	5 (0.4)	0 (0.0)*	1 (0.8)**	4 (9.5)***
Death in-hospital [*n* (%)]	51 (4.0)	19 (1.7)*	16 (12.0)**	16 (38.1)***
30-day-mortality [*n* (%)]	50 (3.9)	18 (1.6)*	16 (12.0)**	16 (38.1)***
LOS ED (min, median, IQR)	374 (205–693)	340 (188–597)*	718 (438–1284)**	539 (292–779)***
LOS ICU (days, median, IQR)	3 (2–7)	3 (1–10)	3 (2–5.75)	5 (2–8)
LOS hospital (days, median, IQR)	0 (0–8)	0 (0–6.0)*	9 (0–6)	5.5 (0–15.0)** *

*n*, number; ICU, intensive care unit; ED, emergency department; LOS, length-of-stay; min, minutes.

*p* is significant, if *p* < 0.05; * = *p*_I,II_ < 0.05; ** = *p*_II,III_ < 0.05; *** = *p*_I,III_ < 0.05.

The bold values represent significant results.

Length of stay (LOS) in the ED for patients with infection, sepsis, and septic shock was 500 ± 505, 867 ± 507, and 666 ± 475 min, respectively. Hospital LOS was 5 ± 9, 12 ± 14, and 11 ± 15 days in groups I–III, respectively.

The 30-day mortality increased significantly from 1.6% in patients with infection, to 12.0 in patients suffering from sepsis and 38.1% from septic shock. As many as four patients with septic shock (9.5%) died in the ED. In contrast, one patient with sepsis (0.8%) and no patient with infection died in the ED. During the whole hospital stay, 1.7% from group I died, as well as 12.0% from group II and another 38.1% from group III.

## Discussion

In the present EpiSEP study, we show for the first time the significant differences in epidemiology, management and outcome of patients with infection alone, sepsis and septic shock in the ED. In the study cohort, which included more than 6,000 ED visits, one in five ED patients suffered from an infection during the study period. Using the SEPSIS-3 definition (6), 10.4% of the patients with infections suffered from sepsis, and 3.3% from septic shock. Our study thus shows for the first time care data and approximate incidence rates of infections, sepsis and septic shock in ED patients.

Despite considerable advances in medicine, sepsis is a condition that continues to be associated with high inpatient mortality, being the third leading cause of death in non-surgical ICU and the leading cause of death in non-cardiac and surgical ICU (17–19). Previous epidemiological studies on sepsis and septic shock were mainly conducted in the ICU setting (9, 18, 20–22) (Table 4).

**TABLE 4:** Comparison of EpiSEP with intensive care unit-studies on epidemiology and causes of infection, sepsis, and septic shock.

Source (1)	EpiSep study 1 ED, Germany			EPIC II 1,265 ICUs in 75 countries (667 ICUs in Western Europe, 2007) (18)	EPIC III 1,150 ICUs in 88 countries (479 ICUs in Western Europe, 2017) (21)	SPICE-ICU 22 ICUs, Japan (20)		INSEP-study 434 ICUs, Germany (9)		MEDUSA 44 ICUs, Germany (22)	
						
	Infection (*n* = 1,103)	Sepsis (*n* = 133)	Septic shock (*n* = 42)	Infection (*n* = 7,087)	Infection (*n* = 8,135)	Sepsis-2 (*n* = 530)	Sepsis-3 (*n* = 569)	Sepsis (*n* = 211)	Septic shock (*n* = 190)	Intervention group (*n* = 2,596)	Control group (*n* = 1,587)
Respiratory tract	362 (34.9)	58 (43.6)	26 (61.9)	4503 (63.5)	4893 (60.1)	200 (37.7)	208 (36.6)	141 (66.8)	111 (58.4)	1078 (41.6)	688 (43.4)
Urogenital tract	193 (17.5)	36 (27.1)	6 (14.3)	1011 (14.3)	1138 (14)	91 (17.2)	101 (17.8)	17 (8.1)	8 (4.2)	314 (12.1)	216 (13.6)
ENT/OMF	178 (16.1)	3 (2.3)	0 (0.0)	n.d.	n.d.	n.d.	n.d.	n.d.	n.d.	n.d.	n.d.
Intra-abdominal	146 (13.3)	16 (12.0)	4 (9.5)	1392 (19.6)	1490 (18.3)	111 (20.9)	119 (20.9)	59 (28.0)	77 (40.5)	974 (35.7)	568 (35.8)
Soft tissue	120 (10.9)	5 (3.8)	1 (2.4)	467 (6.6)	518 (6.4)	5 (0.9)	5 (0.9)	20 (9.5)	16 (8.4)	207 (8.0)	148 (9.3)
Unknown	55 (5.0)	11 (8.3)	4 (9.5)	n.d.	n.d.	22 (4.2)	24 (4.2)	n.d.	n.d.	96 (3.7)	50 (3.3)
Others	31 (2.8)	1 (0.8)	0 (0.0)	540 (7.6)	529 (6.5)	14 (2.6)	16 (2.8)	n.d.	n.d.	19 (0.7)	17 (1.1)
Cerebral	12 (1.1)	2 (1.5)	1 (2.4)	208 (2.9)	314 (3.9)	11 (2.1)	13 (2.3)	n.d.	n.d.	43 (1.7)	22 (1.4)
Blood stream	6 (0.5)	1 (0.8)	0 (0.0)	1071 (15.1)	1239 (15.2)	n.d.	n.d.	n.d.	n.d.	n.d.	n.d. n.d.

ED, emergency department; ICU, intensive care unit; ENT, ear/nose/throat; OMF, oral maxillofacial; n.d., no data.

There, a significant proportion of infections are of nosocomial origin, so that the source of infection is much more frequently determined by previous operations, interventions or prolonged invasive ventilation. Moreover, intensive care patients often require more specific therapeutic measures than patients who present to the ED for the first time with symptoms that may initially be unspecific. In the Extended Prevalence of Infection in Intensive Care (EPIC) I study, showed that 45% of ICU patients were treated due to one or more infections. Of these, only 14% were community-acquired, whereas 10% were hospital-acquired, and 21% ICU-acquired (23). EPIC III came up with similar results in terms of ICU-acquired infections (21). The prospective, multicenter German Incidence of severe sepsis and septic shock (INSEP) study even described that 57% of sepsis cases were nosocomial-associated, and, of these, 50% were ICU-acquired (9). Consequently, these epidemiological figures from studies in the intensive care unit cannot be transferred to the ED. Although according to the recommendations of the Surviving Sepsis Campaign (SSC) guidelines (7) sepsis should be recognized as soon as possible, there are no comprehensive epidemiologic studies on infection, sepsis, and septic shock in the ED.

In a comparison of our epidemiologic data with the most common ICU studies on sepsis (9, 18, 20–22) (Table 4), we were able to show that the weighting of the focal distribution differs significantly apart from the respiratory and genitourinary tracts. It suggests that abdominal and bloodstream infections are significantly more common in ICU than in the ED. This is probably due to nosocomial acquired infections in particular. Although the dominant infection focus in the ED is represented by the respiratory tract, it appears to be a disproportionately frequent focus in EPISEP compared with other ED studies (Table 5). The reason for this could be the seasonal influence in the EpiSEP study. In the EpiSEP study, soft tissue infections also occur significantly less frequently than in all other ED studies included in Table 5, which may be due to the fact that our dermatology department has its own ED. The proportion of unknown infection sites also seems to be lower in ICUs than in ED patients, which is probably due to the more aggressive diagnostics. These differences indicate that sepsis appears to present even more heterogeneously in the ED than in intensive care units. Future guidelines should take this into account in order to optimize early diagnosis and treatment already in the ED.

**TABLE 5:** Comparison of EpiSEP study with studies from emergency department on epidemiology and causes of infection, sepsis, and septic shock.

Source	EpiSEP study 1 ED, Germany			ARISE–study 51 EDs, Australia, New Zealand, Finland, Hong Kong, Republic of Ireland (24)		ProMISE 56 EDs, England (25)		ProCESS-study 31 EDs, United States (26)			Epidemiology of emergency department sepsis data from the National Health Informatics Project, Taiwan (27)		The impact of the Sepsis-3 definition on ICU admission of patients with infection 1 ED, Germany (28)
						
	Infection (*n* = 1,103)	Sepsis (*n* = 133)	Septic shock (*n* = 42)	Septic shock EGDT (*n* = 793)	Septic shock usual care (*n* = 798)	Septic shock EGDT (*n* = 625)	Septic shock usual care (*n* = 626)	Sepsis protocol-based EGDT (*n* = 439)	Sepsis protocol-based standard-therapy (*n* = 446)	Sepsis usual care (*n* = 456)	Sepsis ED admitted (*n* = 493,397)	Sepsis non-ED-admitted (*n* = 763,287)	Infection (*n* = 916)
Respiratory tract	362 (32.8)	58 (43.6)	26 (61.9)	289 (36.5)	262 (32.8)	228 (36.5)	207 (33.1)	140 (31.9)	152 (34.1)	151 (33.1)	277,945 (56.3)	398,504 (52.2)	(56.8)
Urogenital tract	193 (17.5)	36 (27.1)	6 (14.3)	148 (18.7)	160 (20.1)	108 (17.3)	117 (18.7)	100 (22.8)	90 (20.2)	94 (20.6)	193,060 (39.1)	234,313 (30.7)	(24.6)
ENT/OMF	178 (16.1)	3 (2.3)	0 (0.0)	n.d.	n.d.	n.d.	n.d.	n.d.	n.d.	n.d.	n.d.	n.d.	n.d.
Intraabdominal	146 (13.2)	16 (12.0)	4 (9.5)	63 (8.0)	61 (7.6)	40 (6.4)	51 (8.1)	69 (15.7)	57 (12.8)	51 (11.2)	32,082 (6.5)	41,052 (5.4)	(7.5)
Soft tissue	120 (10.9)	5 (3.8)	1 (2.4)	90 (11.4)	76 (9.5)	39 (6.2)	39 (6.2)	25 (5.7)	33 (7.4)	38 (8.3)	34.058 (6.9)	28,931 (3.8)	(5.6)
Unknown	55 (5.0)	11 (8.3)	4 (9.5)	52 (6.6)	72 (9.0)	76 (12.2)	77 (12.3)	57 (13.0)	47 (10.5)	66 (14.5)	n.d.	n.d.	n.d.
Others	31 (2.8)	1 (0.8)	0 (0.0)	52 (6.6)	72 (9.0)	21 (3.4)	37 (5.9)	28 (6.4)	31 (7.0)	26 (5.7)	n.d.	n.d.	(5.5)
Cerebral	12 (1.1)	2 (1.5)	1 (2.4)	13 (1.6)	6 (0.8)	12 (1.9)	9 (1.4)	3 (0.7)	3 (0.7)	4 (0.9)	n.d.	n.d.	n.d.
Blood stream	6 (0.5)	1 (0.8)	0 (0.0)	75 (9.5)	86 (10.8)	97 (15.5)	86 (13.7)	n.d.	n.d.	n.d.	n.d.	n.d.	n.d.

ED, emergency department; ICU, intensive care unit; ENT, ear/nose/throat; OMF, oral maxillofacial; n.d., no data.

As shown in Table 5, sepsis and septic shock seem to be very heterogeneous regarding the source of infection in the ED (24–28). Different approaches to identify patients with sepsis and septic shock confound the true incidence of these conditions. This is often based on now outdated sepsis definitions or on the inclusion of a study population based on ICD-10 coding. Overall, the respiratory tract clearly dominates in septic shock in the study comparison (24–28). Nevertheless, the question remains as to where the differences in the source of infection between EpiSEP (61.9% respiratory tract in septic shock) and, for example, ARISE (32.8% respiratory tract in septic shock) originate (24).

With reference to the epidemiological findings of the EpiSEP study, patients of the infection group were younger than these suffering from sepsis and septic shock, whereas the mean age of the latter was the same or older such as in the EGDT River’s study (29), ARISE (63 ± 17) (24), ProCESS (60 ± 16) (26), and ProMISE (66 ± 15) (30).

Consistent with the results of other studies (22–25), patients in the EpiSEP study showed significant changes in vital signs as a function of disease severity: Compared with patients with infection alone, patients with sepsis or septic shock were more hypotensive, presented with tachycardia, and had a higher respiratory rate as well as lower shock index and oxygen saturation. In line with the patients suffering from septic shock in the ARISE study (24), patients of the EpiSEP study with septic shock showed a comparable mean lactate level of 4.4 ± 3.8 mmol/l.

The group comparison showed a significant discrimination of the three groups by the emergency medicine sepsis scores (Table 1). The SOFA score used by the guideline for the detection of sepsis is not immediately available at the time of admission due to parameters such as the Horovitz quotient or necessary laboratory values. Furthermore, at least 21.8% in group II and 7.1% of the patients with a septic shock arrived in the ED as a “walking emergency.” This shows the importance of a structured assessment and the use of scores to recognize critically ill patients at ED admission.

We found that the infection sites in the three subgroups of the EpiSEP study differ significantly. The leading causes of infection in the EpiSEP study were respiratory tract disease, genitourinary tract disease, maxillofacial/ears/nose/throat area and intra-abdominal causes, and soft tissue infections. The high proportion of patients with infection focus in the maxillofacial/ears/nose/throat area is the prime example that these focuses are very relevant in an ED but does not seem to represent a relevant focus for sepsis and septic shock. In addition, there are more patients in the ED whose infectious focus could not be clearly identified during the ED stay.

Patients with severe sepsis and septic shock in the four mentioned ED studies showed the following causes: respiratory tract 31.9–39.5%, urogenital tract 17.3–27.2%, intraabdominal 5.9–15.7%, and other causes in 26.9–40.0% (22–25). Based on a comparison of different ICU studies (Table 5), with increasing disease severity the respiratory tract is the dominant focus in sepsis and septic shock, the other causes are more or less comparable (9, 18, 20–22).

The 1-h bundle of the SSC included (1) measurement of lactate level, (2) collection of blood culture before administration of antibiotics, (3) early administration of broad-spectrum antibiotics, (4) initiation of rapid administration of crystalloid solution, (5) application of vasopressors (7). While these 5 items were not fulfilled or only partially fulfilled in the infection group, the degree of fulfillment was higher in the EpiSEP group with sepsis and septic shock. The chosen time of measurement, 1 h after admission, suggests that the actual guideline adherence with fulfillment 1 h after diagnosis, should be significantly higher. Nevertheless, future timely documentation is essential for accurate evaluation of guideline adherence.

As recommended by SCC, measurement of lactate in the EpiSEP study was performed in more than 94.7–95% in patients suffering from sepsis and septic shock (Table 2). Positive blood cultures are associated with more frequent multiorgan failure and higher mortality. Therefore, the obligatory recruitment of blood cultures in the ED with subsequent possible isolation of a pathogen sets the course for an empirical adjustment of antibiotic therapy during the course (31). The compliance of our ED treatment with the SSC guidelines in the subgroup of patients suffer from sepsis and septic shock can be considered as very high as the proportion of performed blood cultures was 81–88%, and the administration of broad-spectrum antibiotics after blood culture recruitment was performed in 71–76% of cases. It is well known that the initial administration of broad-spectrum antibiotics, must be reevaluated promptly in the early follow-up. Antibiotic administration occurred within 1 h in 8.3% in sepsis and 14.3% in septic shock (Table 2). The validity of these data is limited by the time of measurement that was chosen (1 h after admission). Even assuming that guideline adherence would be significantly better if the measurement time point was 1 h after diagnosis in accordance with guidelines, this remains a result to be critically evaluated. The resulting optimization potential must be evaluated in the future by real-time documentation to be able to set the results in relation to guideline adherence.

According to the recently published studies by Permpikul et al. (32) on early vasopressor therapy in septic shock, we administrated vasopressors in a very high percentage of 83.3%.

The relevance of sepsis diagnosis is particularly underpinned in patients with septic shock, as delaying an initiation of treatment significantly reduces the likelihood of patient survival (33), so that initiation of adequate treatment in the ED should also occur as soon as possible. With increasing disease severity, the number of invasive procedures (e.g., central venous access, arterial line) performed also increased in accordance with the literature (22–25), although a significantly higher rate of vasopressors and a lower rate of ventilation support were found than in the comparative studies.

In the literature, about one-third of patients entering an ICU are admitted through the ED (21, 28). In the EpiSEP study, half of all ED patients suffering from infection (50.5%) are admitted to the hospital, and the majority (40.5%) were admitted to general wards, only a minority of 5.5% were admitted to ICU. The cases admitted to the normal ward are therefore disregarded in the most common infection and sepsis studies. In our study, 97% of patients with sepsis were admitted as inpatients, but of these only 14.3% went to the ICU. Of the septic shock group, 100% were admitted as inpatients, of which the following proportions were admitted to the normal ward, ICU, or were external transferred: 7, 59.5, 14%, respectively. A total of 9.5% died already in the ED. These data supported the hypothesis that a large proportion of hospital admitted ED patients with sepsis were transferred to the normal ward and are thus excluded from the previous ICU studies. The 30-day mortality rate in the three groups was 1.6, 12.0, and 38.1%, respectively. These findings corresponded to the results of other sepsis studies (22–25).

### Limitations

The major limitation of our investigation is that it is a single-center retrospective study. However, the reliability of the results with over 1,100 patients in group I (infection alone) seems to be sufficiently large. However, the number of patients with sepsis, although identified from an initial cohort of over 6,000 ED patients, appears to be borderline low. Further multicenter studies involving a large number of study centers and a prospective study design should investigate the underlying epidemiology and causes of infection, sepsis and septic shock in the ED setting in a larger cohort. A further limitation is, that due to the local form of organization, some patients are treated in other outpatient clinics (e.g., ophthalmology, dermatology, gynecology), so infections in these patients may be underrepresented in the EpiSEP study cohort. In addition, children, as long as they are not critically ill or injured were treated in the pediatric ED of our institution. In the EpiSEP study these pediatric patients were excluded in order to avoid distortion. Supplementary, elective patients with a possible focus on infection are not included in our study. A further possible bias is that our university hospital is a specialized center for patients suffering from severe diseases (e.g., cancer, hematological diseases) and immunosuppressive state (e.g., heart and kidney transplantation).

Also, one may criticize that the underlying seasonal factors (winter season due to January and February) led to a seasonal bias in the study results, possible overrepresenting respiratory causes of infection, sepsis and septic shock. The validity of the data regarding the 1 h-bundle is limited by the fact that there was no exact time of documentation for the diagnosis “sepsis,” so that we only used values within the first hour after admission to the ED.

## Conclusion

The EpiSEP study shows important care data on patients with infection, sepsis, and septic shock in an German ED. By using vital signs and clinical findings for identification, the study approximates the actual incidence rates of sepsis and septic shock in the ED and emphasizes the importance of sepsis detection and structured diagnosis and therapy.

## Data availability statement

The raw data supporting the conclusions of this article will be made available by the authors, without undue reservation.

## Ethics statement

The studies involving human participants were reviewed and approved by Ethikkommission, Medizinischen Fakultät, Heinrich-Heine-Universität, Düsseldorf, Gebäude 14.82, Ebene 01, Raum 101. Written informed consent for participation was not required for this study in accordance with the national legislation and the institutional requirements.

## Author contributions

NW, MB, and MM conceived the study, analyzed the data, and drafted the manuscript. MB supervised the project as a whole. NW, LB, OH, MB, and MM collected the data. MM, VK, OH, and PK substantively revised the manuscript. All authors read and approved the final version of the manuscript.
